# Supplementary material for: Zinc Protoporphyrin-Rich Pork Liver Homogenates as Coloring Ingredients in Nitrite-Free Liver Pâtés
Source: Foods. 2024 Feb 9;13(4):533. doi: 10.3390/foods13040533 (PMC10887533; doi:10.3390/foods13040533)
Supplement: Supplementary file 1 [file foods-13-00533-s001.zip › foods-2852219-supplementary.pdf]

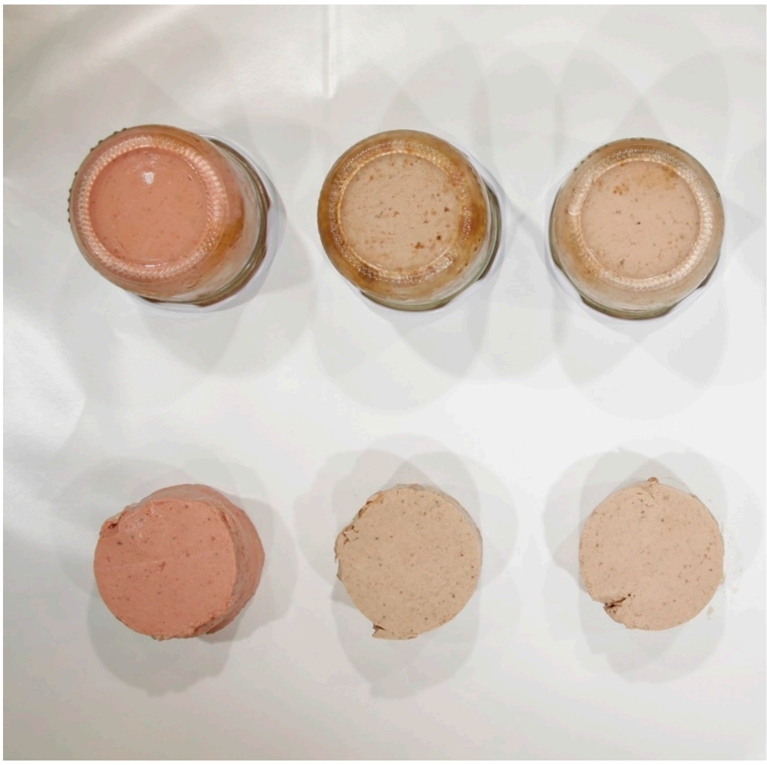

Supplementary Figure S1. Pâtés elaborated with nitrites (left), the same formulation but without the addition of nitrites (center), the full replacement of water by the supernatant without the addition of nitrites (right).
